# Cumulative Fatigue Damage of Composite Laminates: Engineering Rule and Life Prediction Aspect

**DOI:** 10.3390/ma16083271

**Published:** 2023-04-21

**Authors:** Nikolaos D. Batsoulas, Georgios I. Giannopoulos

**Affiliations:** Mechanics and Materials Laboratory, Department of Mechanical Engineering, School of Engineering, University of the Peloponnese, 26334 Patras, Greece

**Keywords:** damage rule, composite materials, composite laminates, fatigue damage accumulation, cumulative fatigue damage, hyperbolic isodamage lines, fatigue life, continuum damage mechanics

## Abstract

The analysis of cumulative fatigue damage is an important factor in predicting the life of composite elements and structures that are exposed to field load histories. A method for predicting the fatigue life of composite laminates under varying loads is suggested in this paper. A new theory of cumulative fatigue damage is introduced grounded on the Continuum Damage Mechanics approach that links the damage rate to cyclic loading through the damage function. A new damage function is examined with respect to hyperbolic isodamage curves and remaining life characteristics. The nonlinear damage accumulation rule that is presented in this study utilizes only one material property and overcomes the limitations of other rules while maintaining implementation simplicity. The benefits of the proposed model and its correlation with other relevant techniques are demonstrated, and a broad range of independent fatigue data from the literature is used for comparison to investigate its performance and validate its reliability.

## 1. Introduction

In engineering structures that use composite materials, fatigue damage is a major cause of failure. The most crucial characteristic for designers and users of composite materials is their ability to withstand fatigue. Composite materials have several advantages over traditional metallic materials, such as higher specific strength and stiffness, particularly in the aerospace industry. Fiber-reinforced composites are generally known to have excellent fatigue resistance. As the number of applied cycles rises, fatigue damage may cause fractures. It is essential to evaluate the damage caused by cycling loading as well as damage accumulation because of changes in amplitude, which is referred to as cumulative fatigue damage. This problem in many of its aspects remains challenging and unclear [1,2,3,4,5,6,7,8,9,10]. When a structural part undergoes repeated mechanical loads, it gradually accumulates damage through a complex sequence of processes such as fiber fracture, matrix cracking, debonding along the fiber-matrix interface, void growth, plus full separation into layers, i.e., delamination. These microscopic aspects of failure under cyclic loading are highly complex, and while there has been progress in understanding the micro-mechanics of fatigue failure, it is mostly used for diagnostic purposes. However, the diversity of service conditions often makes it impossible to obtain design data under identical conditions, so the design of new components for long-term operations is typically based on models. While many fatigue damage rules have been developed, none has received overall acclaim.

The Linear Damage Rule (LDR), also known as the Miner’s Rule or the Palmgren–Miner (P–M) Rule, remains the most commonly used model. The LDR is based on the Palmgren–Miner–Robinson hypothesis of linear summation of damage contributions [11,12,13]. It states that failure occurs when the sum Σn_i_/N_i_ reaches one, where n_i_ represents the number of cycles at σ_i_ stress level, while N_i_ represents the life at this stress level. For two stages, the rule takes the form $n_{1}/N_{1}+n_{2}/N_{2}=1$ (P–M rule). However, this rule often leads to inaccurate and non-conservative life predictions, especially for composite materials under cyclic loading. While the simplicity of the formulations that are based on LDR is attractive for metallic structures, such approximations usually fail to predict the fatigue life of laminated structures due to various reasons that have been thoroughly analyzed elsewhere [14]. Several attempts have been made to create a model by assuming that the accumulation of fatigue damage behaves in a nonlinear manner. An initial attempt to establish a nonlinear model for metals has been made by Marko and Starkey via the introduction of a power rule form [15], expressed by the formula ${\left(n_{1}/N_{1}\right)}^{a_{1}/a_{2}}+n_{2}/N_{2}=1$ for two stages of loading, where the exponent terms a_1_ and a_2_ are functions of the first and second stress levels, respectively. It has been noted that this model is highly adaptable for composite materials [16]. However, determining the exponent’s dependence on stress requires experimental means which is not a simple task. For the Miner Rule, only the S-N curve is necessary. Many theories aim to improve prediction accuracy by adding some information beyond the S-N curve. Numerous nonlinear damage rules have been suggested [4,17,18,19,20,21,22]. Nevertheless, the current theories on cumulative damage were originally developed for metals and may not be entirely suitable for composite materials. Therefore, modifications to these theories are required for composite materials. Various expressions of fatigue damage and cumulative damage have been suggested, with elastic modulus, strength, and fatigue life being the most commonly used factors to assess material damage both qualitatively and quantitatively [23,24,25,26]. However, these models rely heavily on experimental data to estimate important control factors and are difficult to adapt to different conditions [27].

Continuum Damage Mechanics (CDM), introduced by Kachanov and Rabotnov [28,29,30,31], deals with the mechanical behavior of a decaying structure in the continuum scale and gives a better understanding of the fatigue damage process for composite materials [32,33,34,35,36,37,38,39]. In this macroscopic science of continuous damage, the damage variable D is normalized at zero for the initial undamaged state and at unity for the failure (n = N). An advantage of nonlinear models is that they can deal with the effect of the loading sequence, which linear models cannot. Therefore, a nonlinear model of fatigue damage accumulation is formed by selecting and identifying the structure of the function D(n/N). Nonetheless, it is important to note that the major issue of CDM, which is identifying the damage concept and constructing the appropriate evolutionary equation for the damage function, continues to be unresolved. Moreover, it is important to establish a suitable damage growth equation that can accurately reflect the character of the damage in composite materials [40].

The focus of this study is to create a theory capable of predicting the damage and fatigue life of different composite laminates under varying loads. Obviously, there are some differences between composites and metals in terms of fatigue damage. At a macroscopic level, however, composites and metals present distinct similarities regarding their fatigue damage behavior, and these are revealed in the present work with the radical concept of hyperbolic isodamage curves, introduced for the first time by the first author [40]. The theory utilizes CDM and considers remaining life aspects, with failure occurring when the total damage surpasses a critical limit. The resulting fatigue damage accumulation rule is nonlinear but may be straightforwardly applied in design using input data from typical S-N curves. Independent experimental results have confirmed the validity of this model, which offers several advantages over existing approaches. The study concludes with the fact that several well-known fatigue damage rules may be generated from this model as a special case, and it is emphasized that utilizing the proposed model is no more demanding than using the original LDR.

## 2. Damage Function

The accumulation of damage is a phenomenon that relies on a failure path procedure, and its evolution is determined by an equation that involves the damage function D and a number of i = 1, 2, ..., n quantities q_i_ influencing the process of damage accumulation. These quantities q_i_ are dependent on a parameter that characterizes the path of the failure procedure and is denoted as λ. The damage evolutionary equation may be expressed by the following structure [38]:

(1) $$\frac{dD}{d\lambda }=f\left(q_{1}\left(\lambda \right),q_{2}\left(\lambda \right),\ldots ,q_{i}(\lambda )\right)$$

In the occurrence of fatigue in composite laminates, the number of fatigue cycles n is a crucial parameter that characterizes the failure process. Based on the number of cycles n, the equation for the evolution of fatigue damage may be rewritten as:

(2) $$\frac{dD}{dn}=f\left(q_{1}\left(n\right),q_{2}\left(n\right),\ldots ,q_{i}(n)\right)$$

where q_i_ are now defined as functions of n.

For composite laminates fatigue, the above relationship may take the following form [26]:

(3) $$\frac{dD}{dn}=f(D,n,\sigma ,R,f_{n},\xi ,T)$$

where σ denotes the maximum value of stress, R represents the stress ratio, f_n_ stands for the loading frequency, ξ indicates the stacking sequence effect, and, lastly, T symbolizes the temperature, all applied via the same fatigue damage process in a pristine composite medium to create the damage D.

For many failure problems, it is a common tactic to assume that the relative degradation of structural integrity is linearly proportional to the loading evolution. For example, in the case of creep [39], which is a time-dependent phenomenon, it is usually assumed that the relative damage is proportional to the time. Similarly, for the fatigue problem under investigation, which is a cycle-dependent phenomenon, it is realistic to assume that the relative increase in damage is linearly proportional to the relative increase in cycle number. Given such a supposition, the last equation may be reformed as:

(4) $$\frac{dD}{D}=\varphi (\sigma )\frac{dn}{n}$$

where φ is a whole function of factors R, f_n_, ξ, and T. 

A first-order approximation results in a simplified differential equation [39]. Considering that the number of cycles to failure N is related to the maximum stress σ, the stress ratio R, the frequency f_n_, the stacking parameter ξ, and the temperature T as well as that the accumulated damage D reaches the critical threshold D_f_ (D = D_f_) for a particular number of cycles to failure n = N(σ, R, f_n_, ξ, T), the above differential equation is reformed to:

(5) $$\frac{D}{D_{f}}={\left(\frac{n}{N(\sigma ,{R,f}_{n},\xi ,T)}\right)}^{\varphi (\sigma )}$$

Considering that D_f_ = 1 is the critical failure value, the last equation yields the following damage function:

(6) $$D={\left(\frac{n}{N}\right)}^{\varphi (\sigma )}$$

The factor φ(σ) as well as the damage concept still needs to be determined.

The identification of the fatigue damage concept of composite laminates may be achieved by using a curve that associates the loading conditions with the number of cycles to failure. This curve, known as the S-N curve, relates cyclic stress with the number of cycles to failure. In addition, the slope of the S-N curve is affected by the factors of the mean stress, temperature, f_n_, and ξ.

Extensive experimental efforts have been made to determine the relationship between σ and N for various composite laminates and loading conditions. The resulting curve line in a double logarithmic plot is concave downward and does not represent a fatigue limit. This curve can be described by a simple hyperbolic expression [14,41], which is more effective in describing the experimental data than other curves. The expression is given by:

(7) $${\left(log\frac{\sigma }{\sigma_{f}}\right)}^{-1}=c{\left(log\frac{N}{N_{m}}\right)}^{-1}$$

where σ_f_ is the fatigue strength coefficient, i.e., the fatigue strength at one cycle, while N_m_ is the minimum number of cycles that are required in order for the damage to be initiated (number of normalization cycles), and C is a constant. 

**Figure 1:**
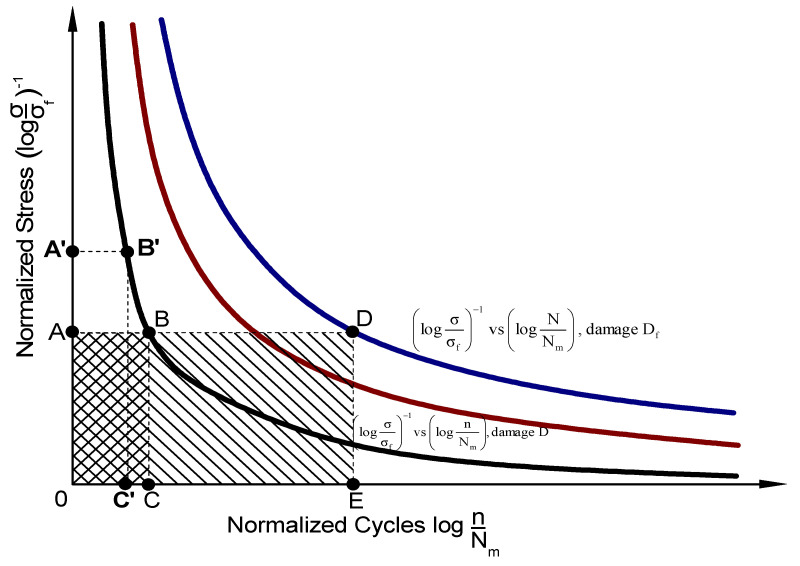
Fatigue damage concept of composite laminates schematically.

Fatigue failure occurs at each point along the [log(σ/σ_f_)]^−1^ vs. log(N/N_m_) curve, while damage occurs without failure at each point along the [log(σ/σ_f_)]^−1^ vs. log(n/N_m_) curve. Each of the three curves, which are depicted in Figure 1, represents one member of an isodamage lines family. As depicted in Figure 1, the upper bound of the fatigue damage zone is defined by the log(σ/σ_f_) vs. log(N/N_m_) curve. In Figure 1, the B and B’ points belong to the [log(σ/σ_f_)]^−1^ vs. log(n/N_m_) curve while the D point lies on the [log(σ/σ_f_)]^−1^ vs. log(N/N_m_) curve. The A point denotes the normalized stress that corresponds to the B as well as D curve points. In addition, the A’ point represents the normalized stress corresponding to the B’ curve point. Finally, the C, E and C’ points indicate the normalized cycles that correspond to the B, D and B’ curve points, respectively. The isodamage curves of this family have a unique feature where their points are vertices of equivalent rectangles. For instance, in Figure 1, the area (0ABC) of the rectangle 0ABC is equal to the area (0A’B’C’) of the rectangle 0A’B’C’ due to Equation (7). The equality of the specific areas is due to the hyperbolic behavior of the isodamage curves, a characteristic that also reflects the experimentally observed hyperbolic damage evolution.

As a result, it is reasonable to classify the fatigue damage concept by means of the magnitude:

(8) $$D=\frac{\left(0ABC\right)}{\left(0ADE\right)}=\frac{{\left(\log\frac{\sigma }{\sigma_{f}}\right)}^{-1}\cdot \log\frac{n}{N_{m}}}{{\left(\log\frac{\sigma }{\sigma_{f}}\right)}^{-1}\cdot \log\frac{N}{N_{m}}}$$

where (0ADE) denotes the area of the rectangle 0ADE. This definition of magnitude D satisfies the boundary conditions D = 1 if n = N as well as D = 0 if n = N_m_.

It should be noted that Equation (6) expresses the damage evolution, while Equation (8) proposes a new approach regarding the fatigue damage concept. However, here, it is proved that the combination of these two expressions, which are certainly not contradictory to each other, may lead to the development of a generalized theoretical model that provides better approximations in comparison with other nonlinear damage rules.

The description of the damage concept by Equation (8) is in agreement with the experimental reality since the S-N curves, independently of their type, are essentially failure curves.

## 3. Damage Analysis under Step Loading

**Figure 2:**
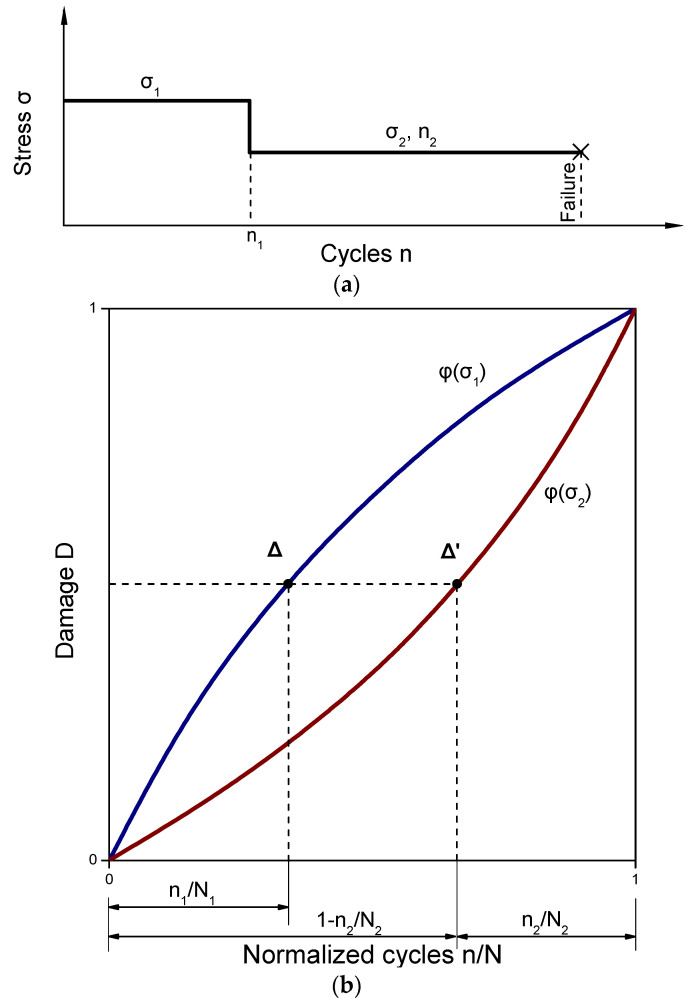
Schematic illustration of fatigue growth until failure: (**a**) loading conditions with respect to cycles; (**b**) fatigue damage with respect to normalized cycles.

Figure 2 illustrates the curves for various exponents φ(σ) based on Function (6). Assume that the (n_1_, σ_1_) conditions are applied during the first step. At the finalization of the first step, the accumulated damage level D can be represented by the point Δ, which corresponds to the life at the σ_1_ level, denoted as N_1_. Moving to the second load level described by (n_2_, σ_2_) conditions, the point Δ shifts to Δ′, where the life N_2_ of the σ_2_ level is located. The loading continues until D equals one, which results in failure. By knowing the damage curves for different φ(σ) values, the lives can be estimated. As the damage defined by points Δ and Δ′ in Figure 2b is the same, the following equation can be derived:

(9) $${\left(\frac{n_{1}}{N_{1}}\right)}^{\varphi (\sigma_{1})}={\left(1-\frac{n_{2}}{N_{2}}\right)}^{\varphi (\sigma_{2})}$$

or

(10) $${\left(\frac{n_{1}}{N_{1}}\right)}^{\varphi_{1,2}}+\frac{n_{2}}{N_{2}}=1$$

where

(11) $$\varphi_{1,2}=\frac{\varphi (\sigma_{1})}{\varphi (\sigma_{2})}$$

Equation (10) may take the form:

(12a) $$\frac{n_{2}}{N_{2}}=1-{\left(\frac{n_{1}}{N_{1}}\right)}^{\varphi (\sigma_{1})/\varphi (\sigma_{2})}$$

From this equation, it is seen that the term (n_1_/N_1_)^φ(σ_1_)/φ(σ_2_)^ is an equivalent cycle ratio at the second stress level, due to a cycle ratio n_1_/N_1_ run at the first stress level. Therefore, the total number of equivalent cycles n_2q_ at the end of the second level is given by:

(12b) $$\frac{n_{2q}}{N_{2}}={\left(\frac{n_{1}}{N_{1}}\right)}^{\varphi (\sigma_{1})/\varphi (\sigma_{2})}+\frac{n_{2}}{N_{2}}$$

We can now treat the equivalent number of cycles n_2q_ at σ_2_ and n_3_ at σ_3_ as a two-stage loading. It is:

(12c) $${\left(\frac{n_{2q}}{N_{2}}\right)}^{\varphi (\sigma_{2})/\varphi (\sigma_{3})}+\frac{n_{3}}{N_{3}}=1$$

Substituting Equation (12b) into Equation (12c), we obtain the following relationship:

(12d) $${\left({\left(\frac{n_{1}}{N_{1}}\right)}^{\varphi (\sigma_{1})/\varphi (\sigma_{2})}+\frac{n_{2}}{N_{2}}\right)}^{\varphi (\sigma_{2})/\varphi (\sigma_{3})}+\frac{n_{3}}{N_{3}}=1$$

or

(12) $${\left({\left(\frac{n_{1}}{N_{1}}\right)}^{\varphi_{1,2}}+\frac{n_{2}}{N_{2}}\right)}^{\varphi_{2,3}}+\frac{n_{3}}{N_{3}}=1$$

where n_1_/N_1_ and n_2_/N_2_ are the cycle ratios that hold for the first and second steps, respectively, n_3_/N_3_ is the estimated remaining cycle ratio that corresponds to the final third step, and φ_2,3_ = φ(σ_2_)/φ(σ_3_).

Evidently, when a multistep loading is considered, then the equivalent form of the aforementioned equation is:

(13) $${\left(\ldots {\left({\left({\left(\frac{n_{1}}{N_{1}}\right)}^{\varphi_{1,2}}+\frac{n_{2}}{N_{2}}\right)}^{\varphi_{2,3}}+\frac{n_{3}}{N_{3}}\right)}^{\varphi_{3,4}}+\frac{n_{\nu -1}}{N_{\nu -1}}\right)}^{\varphi_{\nu -1,\nu }}+\frac{n_{\nu }}{N_{\nu }}=1$$

where subscript ν denotes the final step, while:

(14) $$\varphi_{\nu -1,\nu }=\frac{\varphi (\sigma_{\nu -1})}{\varphi (\sigma_{\nu })}$$

Note that the above exponent φ_ν−1,ν_ is yet to be determined.

**Figure 3:**
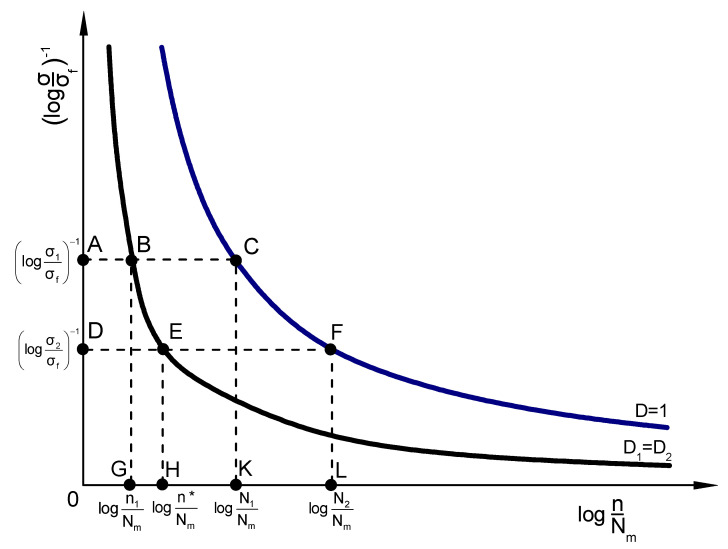
Schematic representation of fatigue damage accumulation regarding a two-stage loading.

Let us assume a composite specimen under a loading defined by the (n_1_, σ_1_) conditions. The damage D is equal to the ratio of the (ABGO) area to the (ACKO) area, both depicted in Figure 3. In the specific figure, the B and E points belong to the curve for D_1_ = D_2_ while the C and F points lie on the curve for D = 1. The A point denotes the normalized stress that corresponds to the B as well as C curve points whereas the D point denotes the normalized stress that corresponds to the E as well as F curve points. Finally, the G, H, K, and L points indicate the normalized cycles that correspond to the B, E, C and F curve points, respectively. Moreover, assume that the failure occurs after a second loading step governed by the (n_2_, σ_2_) conditions. The cycles n_2_ at the second step may be expressed as:

(15) $$n_{2}=N_{2}-n^{*}$$

where n* represents the number of additional cycles that are required within the second load step in order to yield an amount of damage that is equivalent to the one corresponding to the first step (n_1_, σ_1_). Now, Equation (15) may take the form:

(16) $$\frac{n^{*}}{N_{2}}+\frac{n_{2}}{N_{2}}=1$$

The following equations may be obtained from Figure 3 regarding the areas (ABGO) and (DEHO) shown in Figure 3, respectively:

(17a) $$\left(ABGO\right)={\left(\log\frac{\sigma_{1}}{\sigma_{f}}\right)}^{-1}\cdot \log\frac{n_{1}}{N_{m}}$$

(17b) $$\left(DEHO\right)={\left(\log\frac{\sigma_{2}}{\sigma_{f}}\right)}^{-1}\cdot \log\frac{n_{1}}{N_{m}}$$

Since these two areas are equal, the above equations lead to the following expression:

(18a) $$\log\frac{n^{*}}{N_{m}}=\log\frac{n_{1}}{N_{m}}\cdot \frac{{\left(\log\frac{\sigma_{1}}{\sigma_{f}}\right)}^{-1}}{{\left(\log\frac{\sigma_{2}}{\sigma_{f}}\right)}^{-1}}$$

or

(18b) $$\frac{n^{*}}{N_{m}}=10^{\log\frac{n_{1}}{N_{m}}\cdot \frac{\log\frac{\sigma_{2}}{\sigma_{f}}}{\log\frac{\sigma_{1}}{\sigma_{f}}}}$$

or

(18c) $$\frac{n^{*}}{N_{m}}={\left(\frac{n_{1}}{N_{m}}\right)}^{\frac{\log\frac{\sigma_{2}}{\sigma_{f}}}{\log\frac{\sigma_{1}}{\sigma_{f}}}}$$

In addition, regarding the areas (ACKO) and (DFLO) illustrated in Figure 3, it may be written as:

(19a) $$\left(ACKO\right)={\left(\log\frac{\sigma_{1}}{\sigma_{f}}\right)}^{-1}\cdot \log\frac{N_{1}}{N_{m}}$$

(19b) $$(DFLO)={\left(\log\frac{\sigma_{2}}{\sigma_{f}}\right)}^{-1}\cdot \log\frac{N_{2}}{N_{m}}$$

The areas defined by Equations (19a) and (19b) are equal as well and thus:

(20a) $$\log\frac{N_{2}}{N_{m}}=\log\frac{N_{1}}{N_{m}}\cdot \frac{{\left(\log\frac{\sigma_{1}}{\sigma_{f}}\right)}^{-1}}{{\left(\log\frac{\sigma_{2}}{\sigma_{f}}\right)}^{-1}}$$

or

(20b) $$\frac{N_{2}}{N_{m}}=10^{\log\frac{N_{1}}{N_{m}}\cdot \frac{\log\frac{\sigma_{2}}{\sigma_{f}}}{\log\frac{\sigma_{1}}{\sigma_{f}}}}$$

or

(20c) $$\frac{N_{2}}{N_{m}}={\left(\frac{N_{1}}{N_{m}}\right)}^{\frac{\log\frac{\sigma_{2}}{\sigma_{f}}}{\log\frac{\sigma_{1}}{\sigma_{f}}}}$$

By substituting Equations (18c) and (20c) into Equation (16), the following relationship is derived:

(21) $${\left(\frac{n_{1}}{N_{1}}\right)}^{\frac{\log(\sigma_{2}/\sigma_{f})}{\log\left(\sigma_{1}/\sigma_{f}\right)}}+\frac{n_{2}}{N_{2}}=1$$

By comparing Equations (10) and (21), it is concluded that:

(22) $$\varphi_{1,2}=\frac{\log(\sigma_{2}/\sigma_{f})}{\log\left(\sigma_{1}/\sigma_{f}\right)}$$

Generalization of the above equation leads to:

(23) $$\varphi_{\nu -1,\nu }=\frac{\log(\sigma_{\nu }/\sigma_{f})}{\log\left(\sigma_{\nu -1}/\sigma_{f}\right)}$$

Hence, Equation (12) for multistep loading may be written in a generalized form expressing a damage accumulation rule for multilevel fatigue:

(24) $$\begin{array}{c} {\left(\ldots {\left({\left({\left(\frac{n_{1}}{N_{1}}\right)}^{\frac{\log(\sigma_{2}/\sigma_{f})}{\log\left(\sigma_{1}/\sigma_{f}\right)}}+\frac{n_{2}}{N_{2}}\right)}^{\frac{\log(\sigma_{3}/\sigma_{f})}{\log\left(\sigma_{2}/\sigma_{f}\right)}}+\frac{n_{3}}{N_{3}}\right)}^{\frac{\log(\sigma_{4}/\sigma_{f})}{\log\left(3/\sigma_{f}\right)}}+\ldots +\frac{n_{\nu -1}}{{Ν}_{\nu -1}}\right)}^{\frac{\log(\sigma_{\nu }/\sigma_{f})}{\log\left(\sigma_{\nu -1}/\sigma_{f}\right)}}+\\ \frac{n_{\nu }}{N_{\nu }}=1 \end{array}$$

From the arisen generalized rule (24), it becomes evident that the damage accumulation is not solely based on the stress applied but also on the order in which the loading conditions occur.

## 4. Results and Discussion

Rule (24) shares a similar functional form with several well-known fatigue damage rules commonly used in composite materials. Some of these rules can actually be derived as a specific case of formula (24). For instance, LDR may be obtained by using the simplified condition φ_ν−1,ν_ = 1 in Equation (24). Additionally, by setting $\varphi_{\nu -1,\nu }=\alpha_{2}/\alpha_{1}$, where the exponent is determined experimentally [16], we obtain the power rule proposed by Marko and Starkey. Similarly, assuming $\varphi_{\nu -1,\nu }={\left({Ν}_{\nu -1}/{Ν}_{\nu }\right)}^{0.4}$, we obtain the rule suggested by Manson and Halford [42]. Incorporating $\varphi_{\nu -1,\nu }=(1-S_{\nu })/(1-S_{\nu -1})$, where S_ν_ = σ_ν_/σ_u_ and S_ν−1_ = σ_ν−1_/σ_u_, where σ_u_ is the tensile static strength, we obtain the rule proposed by Hashin and Rotem [16,43]. Lastly, utilizing $\varphi_{\nu -1,\nu }=\log\left(w_{\nu }^{t}\right)/log(w_{\nu -1}^{t})$, where w^t^ is the total strain energy density, we obtain the rule proposed by Golos and Ellyin [19].

Besides the above, values to the exponent have been defined in many different ways: entirely experimentally or involving fitting parameters to be determined from experiments [44,45,46,47,48]. It should be noted that the proposed model is grounded on a unique property, i.e., σ_f_, which reflects the material dynamic characteristics. This feature of the presented rule is the main advantage against the other ones, which almost always employ more parameters, some of which result from sophisticated experiments. 

**Figure 4:**
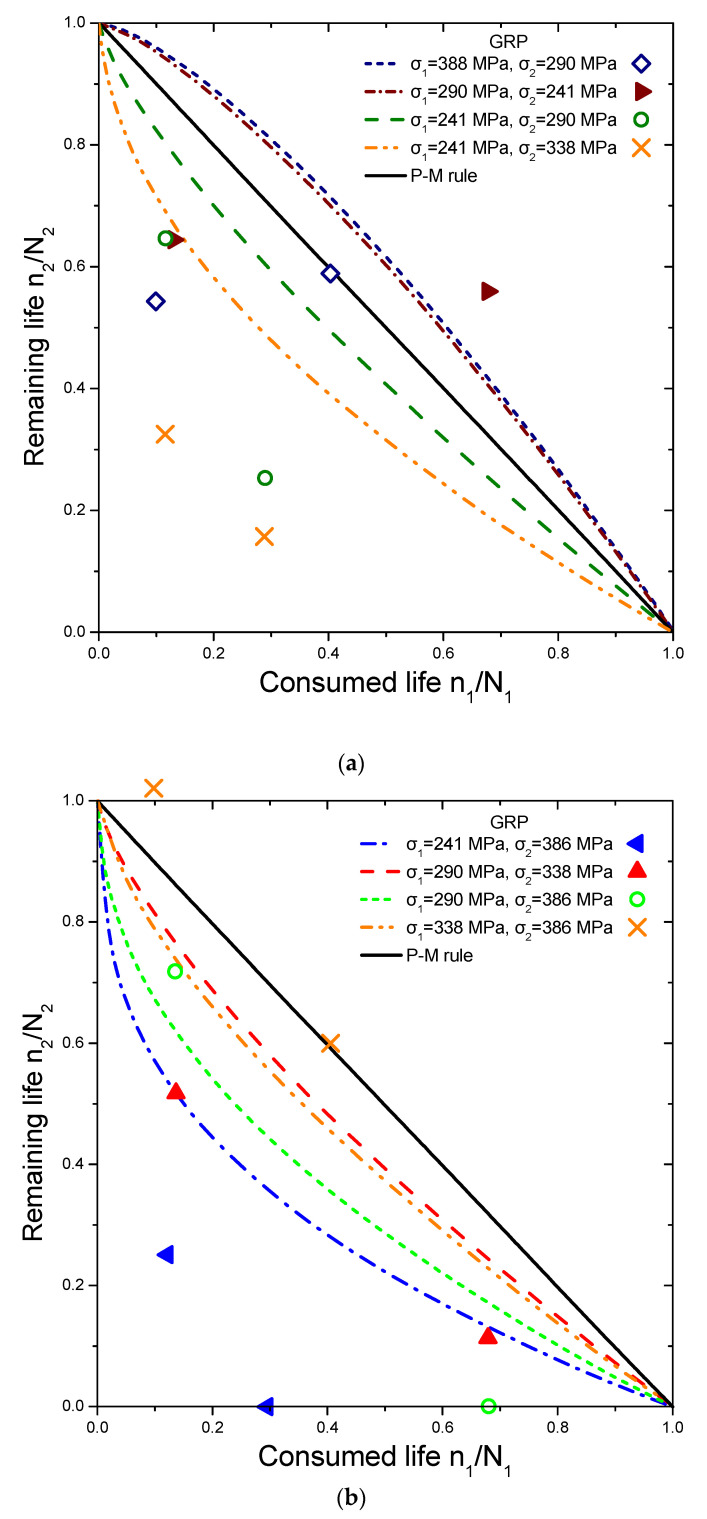
(**a**,**b**) Analytical prediction of the remaining life versus consumed life for the case of glass/epoxy (GRP). The experimental data points are taken from Ref. [44].

**Figure 5:**
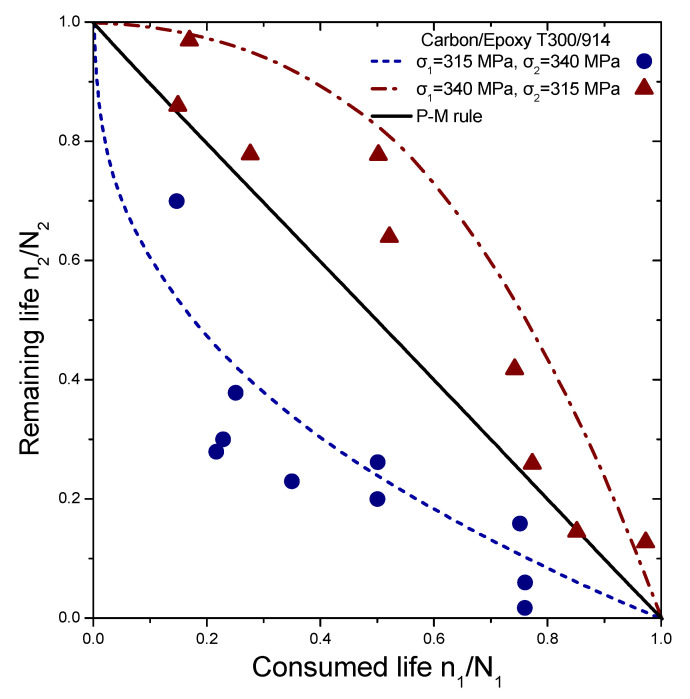
Analytical prediction of remaining life versus consumed life for the case of carbon/epoxy (T300/914). The experimental data points are taken from Ref. [16].

**Figure 6:**
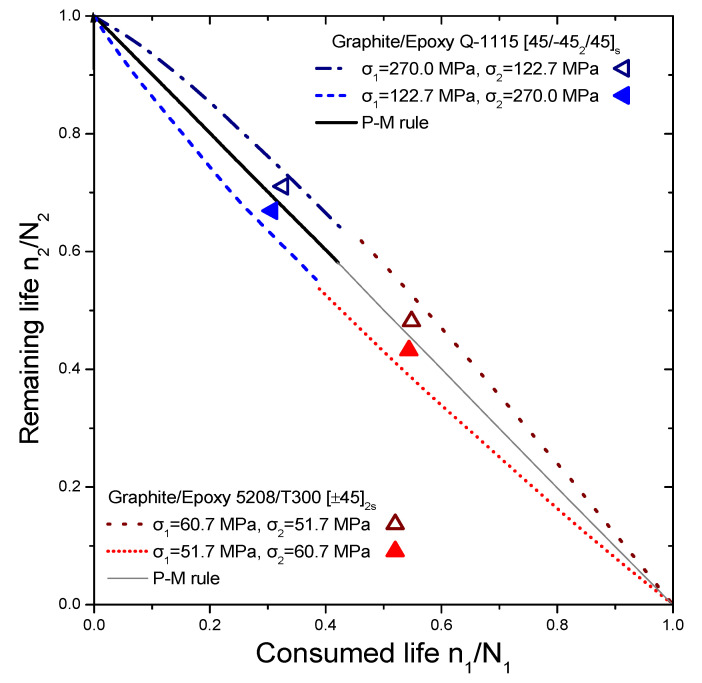
Analytical prediction of remaining life versus consumed life for the case of graphite epoxy (T300/5208) and graphite epoxy (G-1115). The experimental data points are taken from Refs. [49,50].

**Figure 7:**
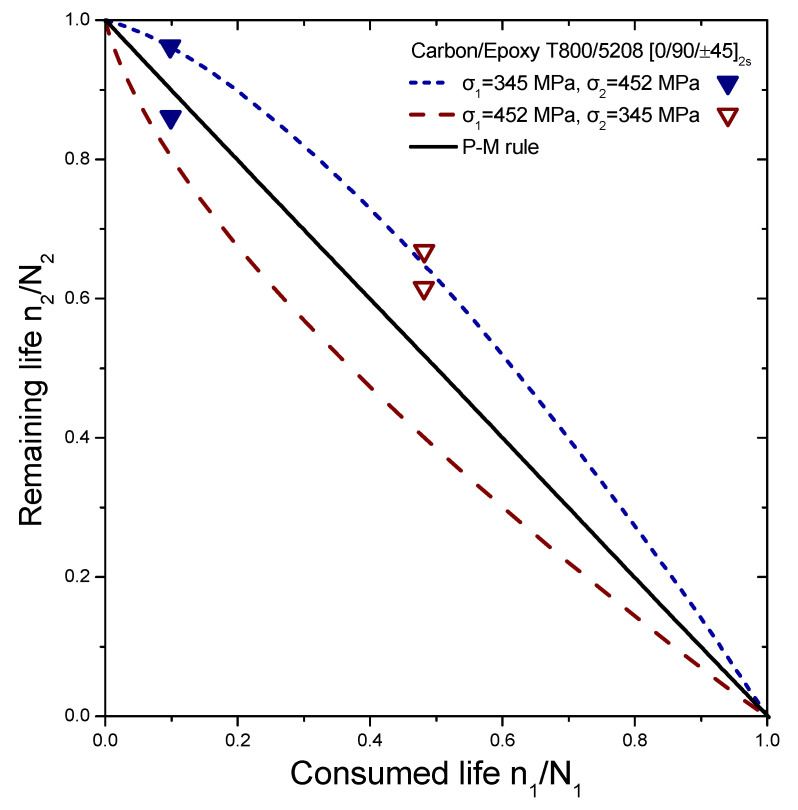
Analytical prediction of the remaining life versus consumed life for the case of carbon/epoxy (T800/5208). The experimental data points are taken from Ref. [41].

The reliability of the proposed model is tested by comparing its prediction results with independent experimental data from different composite laminates and loadings, demonstrating its versatility. This data set contains various experiments conducted on different types of laminates, including cross-ply glass/epoxy (GRP), woven carbon fibre/epoxy resin (carbon-epoxy T300/914C), graphite/epoxy (5208/T300 and T300/5208), and composite laminates (Q-1115). The experiments involve step loadings and stress ratios, with the results presented in Figure 4, Figure 5, Figure 6 and Figure 7. The first step of loading involves n_1_ cycles at stress σ_1_ normalized by lifetime N_1_, while the second one involves σ_2_ with remaining lifetime n_2_ normalized by lifetime N_2_.

The selected experiments used for comparisons include data under step loadings for cross-ply glass/epoxy laminates (GRP) [44], [0/90/±$45_{2}$/10/90]_S_ quasi-isotropic woven carbon fibre/epoxy resin laminate (carbon-epoxy T300/914C) at stress ratio R = 0.05 [16], T300/5208 graphite/epoxy [$\pm 45_{2}$] laminates at stress ratio R = 0.1 [49], T800/5208 graphite/epoxy quasi-isotropic laminates of [0/90/$\pm 45_{2}$]_S_ at stress ratio R = 0.05 [41], and graphite/epoxy (Q-1115) of eight-layer [45/−45_2_/45]_S_ composite laminates [50]. The criterion for the selection of the experimental data was to cover a wide range of composite materials in order to demonstrate the generality of the model and its applicability to treat significantly different materials. The values of the fatigue strength coefficient σ_f_ of all the materials under investigation are presented in Table 1. Generally, it is realistic to assume a 5% variance regarding the estimation of these values. Details about the type of loading may be found in Table 2. The curves shown in Figure 4, Figure 5, Figure 6 and Figure 7 depict the predictions of Equation (21). The P–M rule-based predictions are included in all the following figures for reasons of qualitative comparison. Relatively good agreement between the present prediction and the experimental results may be noticed. The current rule requires the same input as any cumulative damage analysis, and no additional data are needed. All the above results point out that for two-stage loading, a low-to-high loading sequence is generally more damaging than a high-to-low sequence. For many metallic materials, the reverse situation occurs in terms of the sequence of the two-stage loading. In a few cases, only a more damaging effect of the high-to-low sequence has been reported for composite laminates. In these cases, the S-N curves are concave upward, and the rule takes the form [40]:

(25) $${\left(\frac{n_{1}}{N_{1}}\right)}^{\frac{\log(\sigma_{1}/\sigma_{f})}{\log(\sigma_{2}/\sigma_{f})}}+\frac{n_{2}}{N_{2}}=1$$

**Figure 8:**
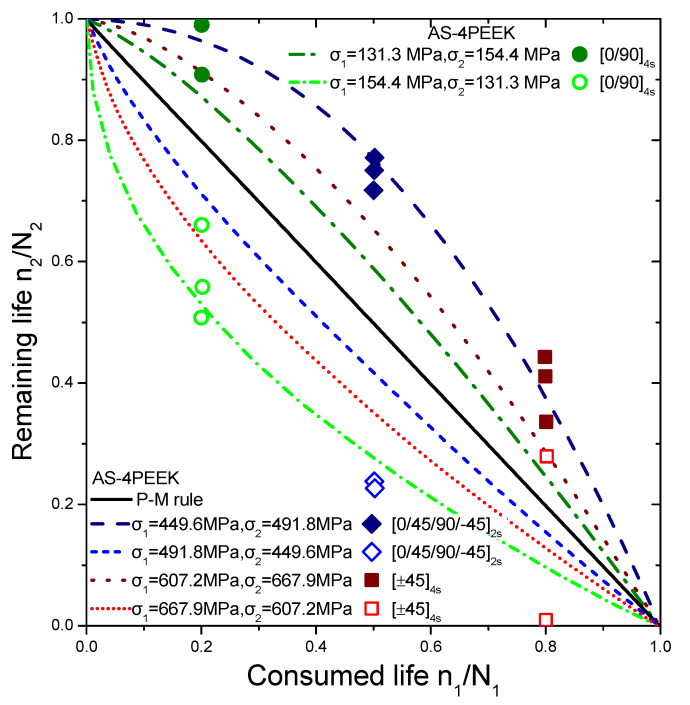
Remaining life versus consumed life analytical predictions for the case of AS-4/PEEK composite laminates. The experimental data points are taken from Ref. [45].

In these cases also, the selected experiments provide measurements under step loading concerning continuous carbon fibers reinforced PEEK thermoplastic matrix composite (AS-4/PEEK). The specific composite material has three layups of [0,90]_4S_, [0/45/90/−45]_2S,_ and [±45]_4S_, while its performance is tested under two stress ratios, R_1_ = 0 and R_2_ = 0.2, and two frequencies, f_1_ = 25 Hz and f_2_ = 5 Hz [45]. The curves that are depicted in Figure 8 are graphical representations of Equation (25). The reasonable agreement between the present results and experimental ones may be seen here as well. A discrepancy may be observed between the present theoretical and other corresponding experimental estimations regarding the GRP case. However, due to the inherent significant variance of the experimental measurements regarding GRP composites, additional experimental evidence combined with statistical analysis is required in future work to reach more coherent conclusions and find the exact limitations of the proposed model. Evidently, more complex loadings including triaxial stress state problems require further investigation.

## 5. Conclusions

This study focuses on a new damage mechanics theory for evaluating fatigue damage and predicting the fatigue life of composite laminates under varying load conditions. The theory is grounded on the hyperbolic isodamage curve family concept, which encompasses any fatigue loading series. Its analytical structure is developed similarly to S-N curve representations, with a failure criterion that relates isodamage curve areas to the number of cycles until failure. With only one material property, a nonlinear cumulative damage rule is fully specified. Some established fatigue damage rules can be derived as special cases of this model. The proposed cumulative damage rule is dependent on stress and sequence and does not require fitting parameters to be evaluated from experiments. The S-N curve establishment is enough for estimating life in loading histories characterized by multiple levels of stress. Various independent tests have been analyzed, and the model’s predictions have a good correlation with the experimental outcomes.

## 6. Future Developments

Due to its general nature, the demonstrated rule may be proposed and evaluated for the representation of the fatigue behavior of composite laminates reinforced with typical graphitic phases, carbon nanomaterials, or metallic nanoparticles. Furthermore, a very interesting goal for future research is the development of the current formulation for the characterization of fatigue damage, considering triaxial stress state and/or phase anisotropy.

## Figures and Tables

**Table 1:** List of the composite materials under investigation, including the corresponding fatigue strength coefficient values.

Material	σ_f_ (MPa)
GRP	506
Carbon/Epoxy T300/914	450
Graphite/Epoxy T300/5208 [±45]_2s_	94
Graphite/Epoxy Q-1115 [45/−45_2_/45]_s_	300
Carbon/Epoxy T800/5208 [0/90/±45]_2s_	846
AS-4/PEEK [0/90]_4s_	454
AS-4/PEEK [0/45/90/−45]_2s_	752
AS-4/PEEK [±45]_4s_	967

**Table 2:** Loading conditions for the composite materials under investigation.

Applied Stresses (MPa)									
GRP	σ_1_	388	290	241	241	241	290	290	338
	σ_2_	290	241	290	338	386	338	386	386
Carbon/Epoxy T300/914	σ_1_	315	340						
	σ_2_	340	315						
Graphite/Epoxy T300/5208 [±45]_2s_	σ_1_	60.7	51.7						
	σ_2_	51.7	60.7						
Graphite/Epoxy Q-1115 [45/−45_2_/45]_s_	σ_1_	270.0	122.7						
	σ_2_	122.7	270.0						
Carbon/Epoxy T800/5208 [0/90/±45]_2s_	σ_1_	345	452						
	σ_2_	452	345						
AS-4PEEK [0/90]_4s_	σ_1_	131.3	154.4						
	σ_2_	154.4	131.3						
AS-4PEEK [0/45/90/−45]_2s_	σ_1_	449.6	491.8						
	σ_2_	491.8	449.6						
AS-4PEEK [±45]_4s_	σ_1_	607.2	667.9						
	σ_2_	667.9	607.2						

## Data Availability

The data that support the findings of this article are available from the corresponding author, upon reasonable request.
